# Stable-Isotope Differentiation of Mature Ovarian Eggs from Anadromous and Freshwater-Resident *Coilia nasus*

**DOI:** 10.3390/ani16172725

**Published:** 2026-09-02

**Authors:** Tao Jiang, Shubing Lin, Xiubao Chen, Junren Xue

**Affiliations:** 1 Wuxi Fisheries College, Nanjing Agricultural University, Wuxi 214081, China; 2023813032@stu.njau.edu.cn; 2 Freshwater Fisheries Research Center, Chinese Academy of Fishery Sciences, Wuxi 214081, China; chenxb@ffrc.cn (X.C.); xuejunren@ffrc.cn (J.X.)

**Keywords:** *Coilia nasus*, otolith, mature egg, *δ*^13^C, *δ*^15^N, spawning site localization

## Abstract

Anadromous and freshwater-resident *Coilia nasus* frequently share spawning grounds, yet exhibit distinct feeding habits during migration; therefore, we hypothesized that the isotopic profiles of mature eggs from these two ecotypes would differ. In the present study, a total of 102 *C. nasus* individuals were collected from a spawning ground in Poyang Lake, China; among them, 20 mature females with intact ovaries were selected for further analysis. Their migratory history was confirmed by otolith microchemistry, and carbon (*δ*^13^C) and nitrogen (*δ*^15^N) stable isotope ratios of mature eggs were analyzed. Otolith microchemical results showed that some of the 20 females exhibited three Sr/Ca stages (1.51 ± 0.46, 4.79 ± 1.00, and 2.09 ± 0.79), identifying them as anadromous, whereas the remaining females showed stable low Sr/Ca ratios (1.49 ± 0.45), indicating freshwater residency. Both *δ*^13^C and *δ*^15^N values of mature eggs from the anadromous individuals were significantly higher than those from the freshwater-resident individuals (*p* < 0.01, Mann–Whitney *U* test). And the results of linear discriminant analysis (LDA) showed no misclassification. These results demonstrated that *δ*^13^C and *δ*^15^N showed strong discriminatory performance for mature eggs from anadromous and freshwater-resident *C. nasus* within the present dataset. However, because all analyses were performed on mature eggs collected directly from ovaries, this method has not yet been tested on naturally spawned or fertilized eggs in the field. Therefore, future studies should first validate this method on field-collected fertilized eggs to determine its practical efficacy for locating spawning grounds.

## Simple Summary

The two fish populations could be distinguished by the chemical signatures of their eggs: one group migrated from the sea to freshwater to spawn (anadromous), and the other resided in freshwater throughout its life. Mature, unfertilized eggs were collected from the ovaries of female fish that were ready to spawn in Poyang Lake, China. First, otolith microchemistry confirmed which fish were migrants and which were freshwater-resident. The stable isotope ratios of carbon (*δ*^13^C) and nitrogen (*δ*^15^N) in these eggs were then measured. Clear differences emerged: eggs from the migratory females had significantly higher *δ*^13^C and *δ*^15^N values than those from freshwater-resident females. Therefore, based on egg isotope signatures, it could be reliably determined whether an egg originated from a migratory or a freshwater-resident mother. The present study suggests the potential for identifying fertilized eggs or locating spawning grounds in the field. Thus, further validation should be carried out by collecting and analyzing fertilized eggs in the field.

## 1. Introduction

*Coilia nasus* is an anadromous species widely distributed in nearshore waters and major rivers of China, Japan, and Korea [1,2,3,4]. The Changjiang River is a primary distribution area of *C. nasus* in China, which historically extended as far as Dongting Lake. However, overfishing, habitat fragmentation, and destruction of key habitats have led to the apparent disappearance of *C. nasus* from some traditional habitats. This even included the Poyang Lake waters, a traditional spawning ground that was once thought to be inaccessible to them [5]. Alongside this anadromous population, the Changjiang River basin also hosts a widespread freshwater-resident population, *C. brachygnathus*, and a landlocked population, *C. nasus taihuensis* [6,7,8]; these taxa are treated here as ecological populations rather than a set of formally defined taxonomic units, given that their taxonomic status remains debated [9,10,11] and natural hybridization occurs in sympatric waters (e.g., Poyang Lake [12]). This diversity has complicated *C. nasus* resource assessment and key habitat identification and protection. Nonetheless, in 2013 we discovered anadromous *C. nasus* populations in Poyang Lake, identifying an important spawning ground near Lushan City based on the distribution of fully mature adults [13].

Since 2019, fishing for certain wild aquatic organisms in the Yangtze River, including *C. nasus*, has initially been prohibited. Subsequently, starting in 2021, a comprehensive 10-year fishing ban was implemented in the Yangtze River. This has significantly protected the biodiversity of aquatic organisms in the Changjiang River, notably improving the recovery of fish resources [14]. However, key habitats of *C. nasus* in the Changjiang River, such as spawning grounds, have not been fully identified. And the presence of multiple ecological distinct populations of *C. nasus*, including anadromous/freshwater-resident *C. nasus* and *C. brachygnathus*, and landlocked *C. nasus taihuensis*, further complicates surveys and conservation efforts [15].

Both *C. nasus*, with a long supermaxilla, and *C. brachygnathus*, with a short supermaxilla, are found in Poyang Lake [15]. The advancement of tissue microchemistry technology, particularly otolith analysis, has significantly enhanced fish migratory ecology research [16,17]. This method evaluates the varying concentrations of certain “geographical fingerprint” elements in different water bodies. For example, strontium has an abundance of 0.02–2.6, 20–86, and 92–114 μmol/L in freshwater, brackish water, and seawater, respectively [18], leading to significant differences in strontium accumulation in the otoliths of species inhabiting these environments [19]. This ultimately allows otolith analysis to accurately record the life history of fish in these different habitats [20,21]. Consequently, otolith microchemistry is widely used to elucidate fish migration paths, population structure, and population connectivity [22,23,24]. Using this technology, the distribution of anadromous *C. nasus* has been identified in other parts of the Poyang Lake basin and tributaries, including the Ganjiang River [25].

Although otolith microchemistry can effectively identify anadromous individuals in adult fish, and stable isotope analysis has been used to differentiate anadromous and freshwater-resident fish and trace their progeny in other species [26], no method has yet been validated specifically for identifying fertilized eggs of *C. nasus* in the field. Ultimately, distinguishing between anadromous and freshwater-resident fertilized eggs can directly affect the identification and conservation of spawning grounds, resource supplementation, and population reproduction. In teleost fishes, the major constituents of eggs are synthesized in the maternal liver and ovary [27]. Carbon and nitrogen incorporated into proteins and lipids are therefore derived directly from maternal diet and metabolic pools, meaning that egg *δ*^13^C and *δ*^15^N values reflect the maternal foraging environment during oogenesis. For anadromous *C. nasus*, feeding is minimal during upstream migration and resumes only after arrival at the spawning ground [28]. This late feeding likely introduces isotopically distinct carbon and nitrogen sources into developing oocytes, resulting in significantly different *δ*^13^C and *δ*^15^N values in oocytes between anadromous and freshwater-resident individuals, which feed within freshwater food webs throughout their life cycle. Since most fish embryos develop independently within an enclosed egg envelope, relying on compounds deposited within the oocytes during development [29], the microchemical characteristics of fertilized eggs from anadromous populations should significantly differ from those of freshwater-resident populations. Additionally, stable isotope ratios, such as *δ*^13^C and *δ*^15^N, are closely associated with food type and feeding behavior, making them valuable tools for studying fish diets and trophic ecological positions [30,31,32]. Therefore, analysis of these ratios can further enhance the understanding of fish migration patterns [33] and population structure [34,35].

In this study, *δ*^13^C and *δ*^15^N characteristics of mature eggs collected from the ovaries of anadromous and freshwater-resident *C. nasus*, which often share the same spawning grounds, were studied to determine whether isotopic differences exist between the two ecotypes and to provide a basis for future discrimination of field-collected fertilized eggs using this approach.

## 2. Materials and Methods

### 2.1. Materials

**Figure 1:**
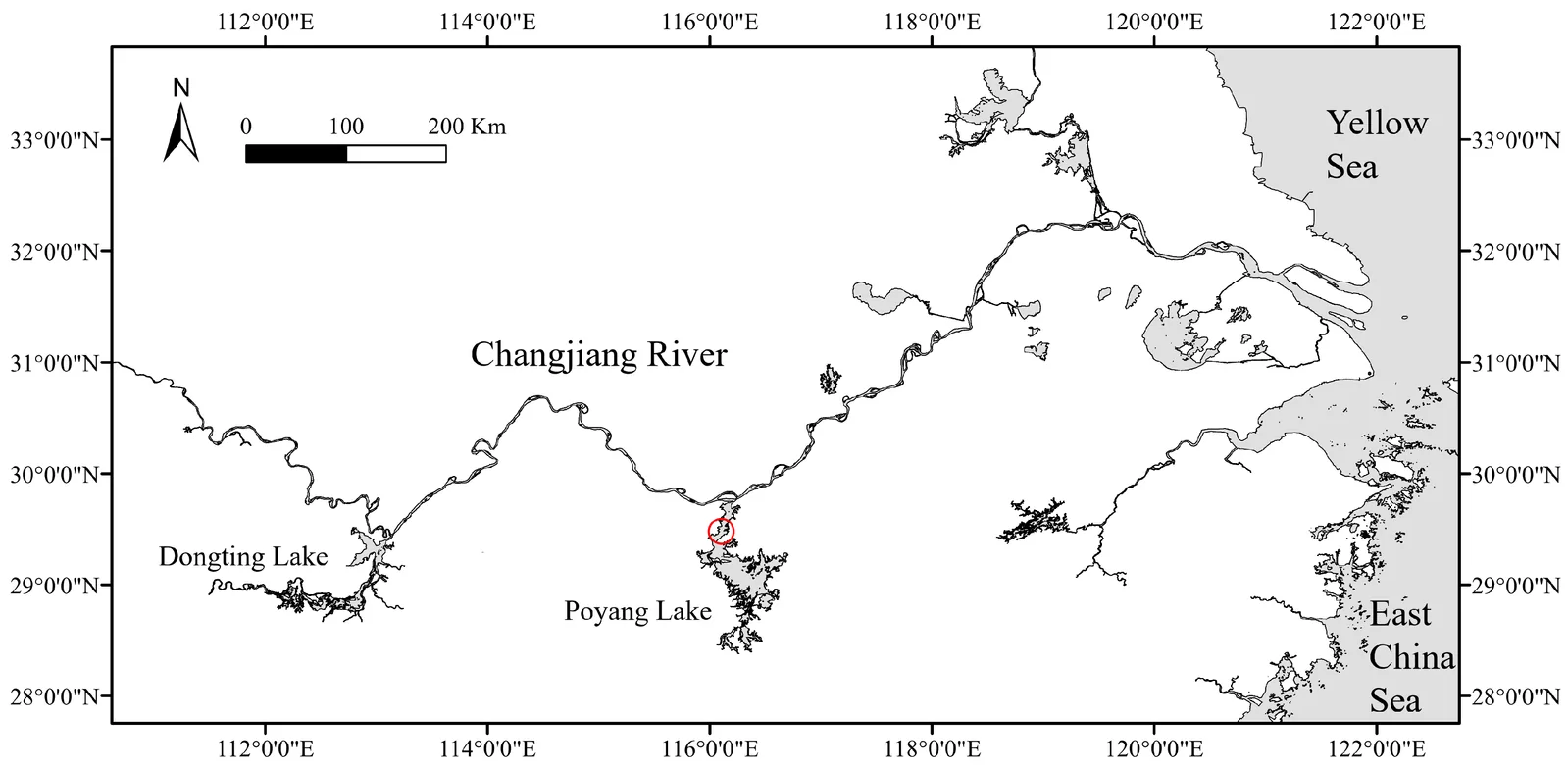
Sampling site in this study; the red circle denotes the region in Poyang Lake where samples were collected.

In July 2023, a total of 102 adult *C. nasus* were collected from Poyang Lake, near Lushan City (116°6′17″ E, 29°29′5″ N), with permission from the local Department of Agriculture and Rural Affairs. Four gill nets, each measuring 50 m in length and 2 m in height with a mesh size of 4 cm, were connected together for sample collection. The nets were set at approximately 15:00 on the first day and retrieved at approximately 05:00 the following morning. Due to their unique behavior, all individuals were dead when removed from the nets. Then, the samples were soon stored at −20 °C and transported to the laboratory (Figure 1). The criteria for determining maturity were based on previous studies, specifically: “Ovaries become grayish, transparent and larger, and cling to no fat tissue. Many mature oocytes are released from their follicles into the ovarian cavity. The oocytes could flow out through the genital opening when the abdomen is gently pressed.” [5]. Among the samples, a total of 20 fully mature females that possessed intact ovaries with a long (*n* = 10, CN) or short (*n* = 10, CB) supermaxilla were selected (Table 1).

### 2.2. Otolith Microchemical Analysis

The left otolith of each fish was extracted, and the Sr/Ca ratio (Sr/Ca × 1000) was analyzed using an X-ray electron probe microanalyzer (JXA-8100 JEOL Ltd.; Tokyo, Japan) with reference to previous studies [25,36]. The accelerating voltage was set to 15 kV, the beam current was set to 20 nA, and the electron beam was focused on a 5-µm diameter point, with measurements spaced at 20-µm intervals. Otolith Sr/Ca ratios were categorized as ≤3, 3–7, and ≥7, corresponding to freshwater, estuarine, and seawater habitats, respectively [37].

### 2.3. Stable C and N Isotope Ratio and Elemental Composition Analysis

Fully mature ovaries were extracted from each fish and washed with Milli-Q water (resistivity 18.2 MΩ·cm at 25 °C; Millipore, MA, USA). The ovaries were then opened, and all eggs from each individual female were pooled together to obtain a composite sample representing that female. The pooled eggs were dried continuously in an oven at 80 °C for 24 h until a constant weight was reached, and were then ground into a uniform powder using an agate mortar. Thus, each female provided a single homogenized sample, yielding one analytical value per female for δ^13^C and δ^15^N. Lipid extraction was not performed in this study, based on previous research [38]. Additionally, to avoid the influence of lipids, both carbon and nitrogen contents were analyzed to correct the *δ*^13^C results.

About 0.2 mg of the powdered egg sample was placed into a tin foil cup (Thermo Fisher Scientific, Waltham, MA, USA). All samples were then analyzed using a stable mass spectrometer (Delta V Advantage, Thermo Fisher Scientific, Waltham, MA, USA) with an elemental analyzer (Flash 2000HT, Thermo Fisher Scientific, Waltham, MA, USA). For mass spectrometry, the carrier gas used was He, with a flow rate of 100 mL/min; the dynamic fast combustion furnace was set to 980 °C, and the column oven was set to 50 °C. Isotope ratios were calculated and expressed using Equation (1):

X = (R_sample_ − R_standard_)/R_standard_ × 1000 (1)

where X denotes *δ*^13^C or *δ*^15^N; R_sample_ represents the corresponding ratio (^13^C/^12^C or ^15^N/^14^N); and R_standard_ denotes the standard for these isotopes, with the standards for *δ*^13^C and *δ*^15^N being Vienna PeeDee Belemnite (VPDB) and atmospheric nitrogen (air N_2_), respectively. During analysis, every 10 samples were interspersed with a standard sample, IAEA600 (*δ*^13^C = −27.771 ± 0.043‰, *δ*^15^N = 1.0 ± 0.2‰, C = 49.4%, N = 28.9%; caffeine, International Atomic Energy Agency, Vienna, Austria), to calibrate the determination results. At the time of determination, the accuracy of continuous determination for *δ*^13^C and *δ*^15^N was <0.03‰. If the IAEA-600 value was outside the reference range, the gas was recalibrated and the sample was re-analyzed.

### 2.4. Statistical Analysis

Lipid correction was performed using the mathematical model from the previous study, as follows:

*δ*^13^C_normalized_ = *δ*^13^C_untreated_ − 3.32 + 0.99 × C:N (2)

where *δ*^13^C_untreated_ is the original *δ*^13^C value, *δ*^13^C_normalized_ is the lipid-corrected value, and C:N is the carbon-to-nitrogen ratio [39]. Hereafter, all instances of *δ*^13^C denote the *δ*^13^C_normalized_ value.

Because the assumption of equal variances was not met (Levene’s test, *p* < 0.05), the non-parametric Mann–Whitney *U* test was used to compare *δ*^13^C and *δ*^15^N between the two ecotypes. In this study, linear discriminant analysis (LDA) with a stepwise method was employed to evaluate whether mature eggs from different ecotypes could be distinguished based on *δ*^13^C and *δ*^15^N signatures. The robustness of the classification model was assessed using a cross-validation test based on the “leave-one-out” procedure. Both statistical analyses were performed using SPSS 29 (IBM Corp., Armonk, NY, USA).

## 3. Results

### 3.1. Determination of Migration Patterns by Otolith Microchemical Characteristics

**Figure 2:**
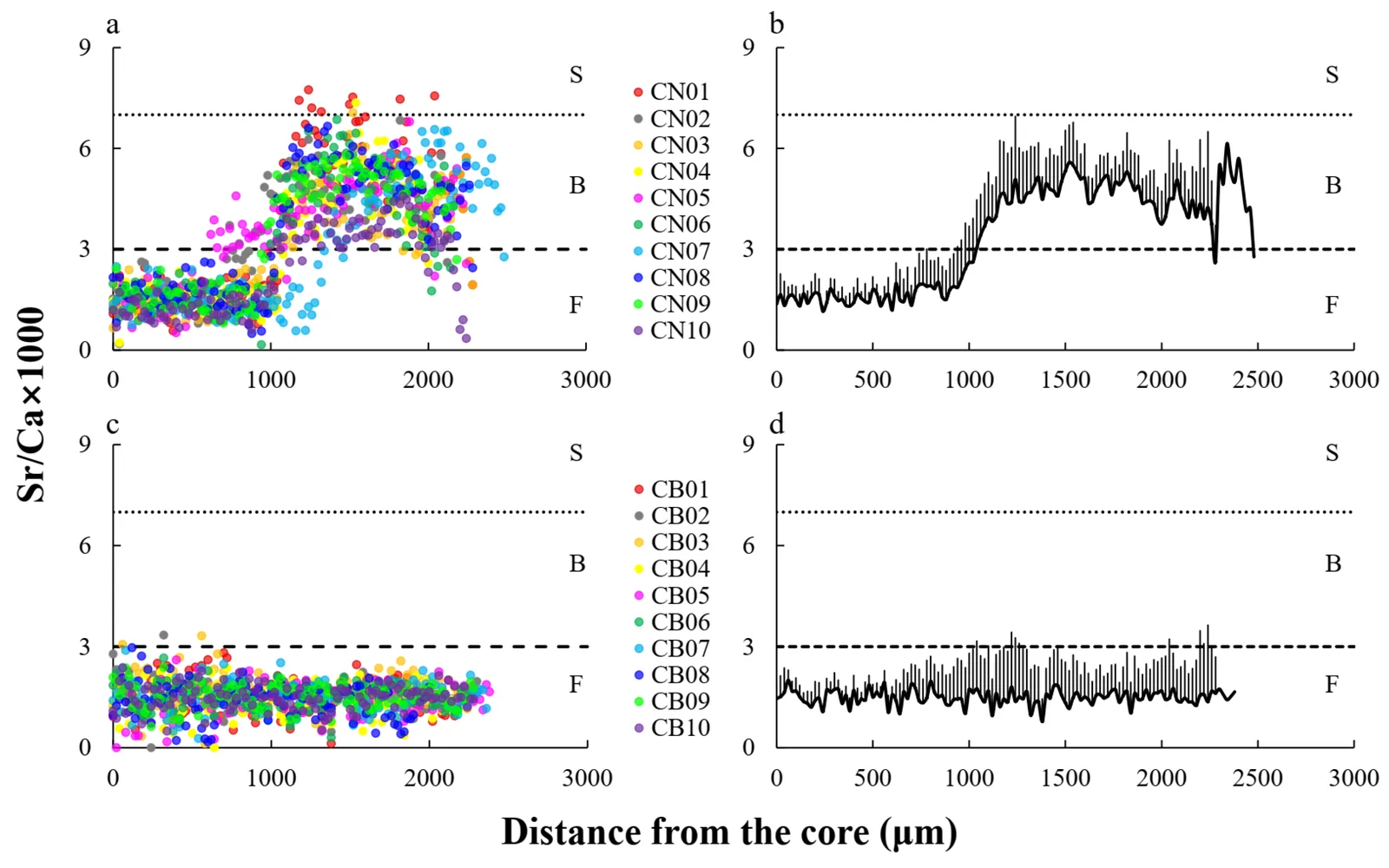
Fluctuations in the Sr/Ca × 1000 values from the core to the edge of otoliths obtained from anadromous and freshwater-resident *Coilia nasus*. (**a**) Scatter plot of individuals with long supermaxillae. (**b**) Average Sr/Ca ratio of individuals with long supermaxillae. (**c**) Scatter plot of individuals with short supermaxillae. (**d**) Average Sr/Ca ratio of individuals with short supermaxillae. Dots and dashed lines represent the threshold values of 7 and 3, respectively. S: Sr/Ca ratios ≥ 7, B: Sr/Ca ratios 3–7, F: Sr/Ca ratios ≤ 3.

The microchemical analysis of otoliths revealed distinct patterns between individuals with long and short supermaxillae. All individuals with long supermaxillae exhibited three stages from the core to the edge of the otolith, with Sr/Ca ratios of 1.51 ± 0.46, 4.79 ± 1.00, and 2.09 ± 0.79 at 620–1360 and 1980–2460 μm from the core, and at the edge, respectively (Figure 2a,b). These stages reflect the classic anadromous life history of *C. nasus* with long supermaxillae. In contrast, all individuals with short supermaxillae showed a stable low Sr/Ca ratio of 1.49 ± 0.45 from the core to the edge, indicative of a classic freshwater-resident life history (Figure 2c,d).

### 3.2. Elemental Compositions and Stable Isotope Analysis of Mature Eggs

**Figure 3:**
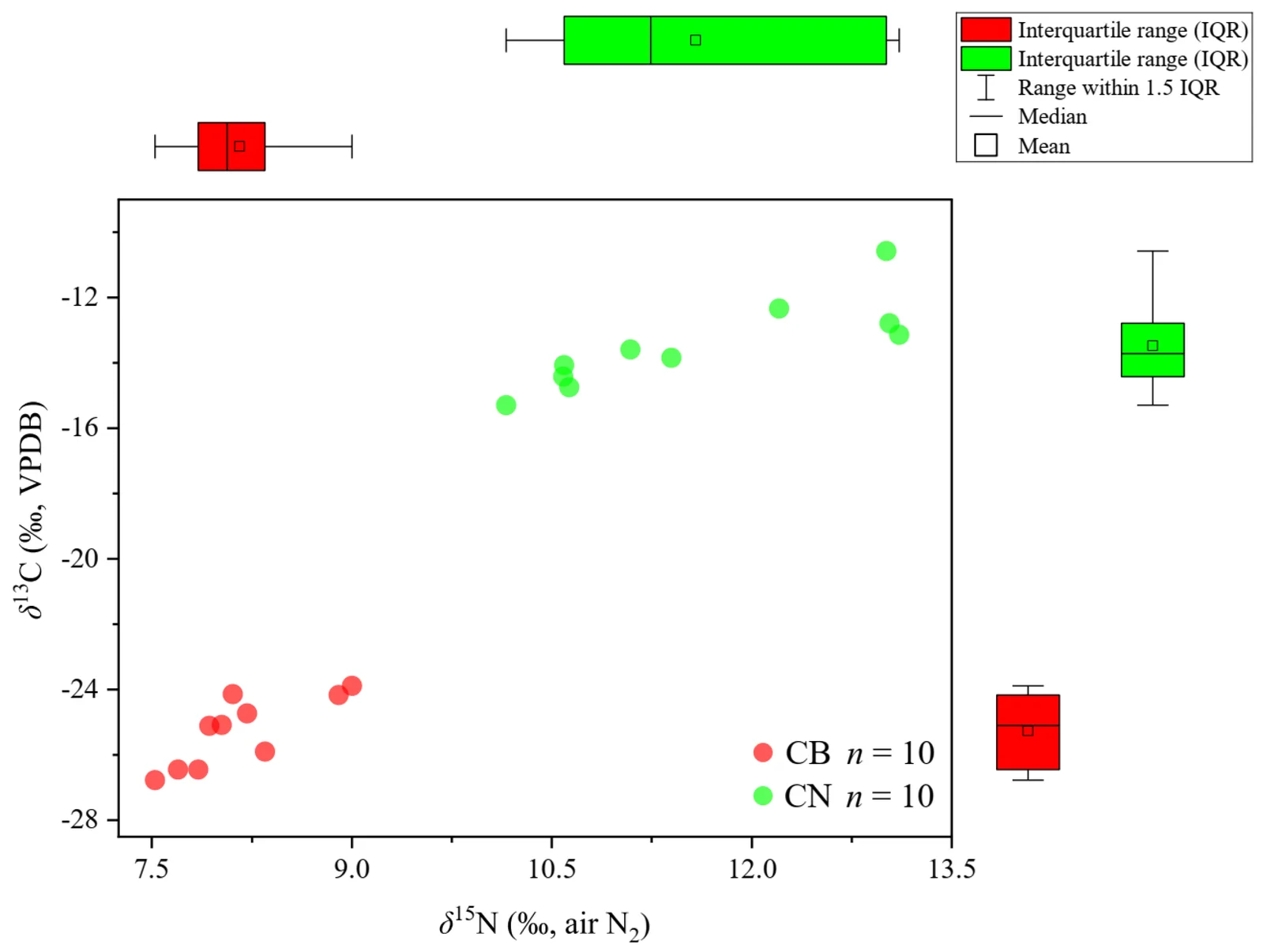
Characteristics of *δ*^13^C and *δ*^15^N in mature eggs obtained from *Coilia nasus*. VPDB: Vienna Pee Dee Belemnite; air N_2_: atmospheric nitrogen; CB: the individual with a short supermaxilla; CN: the individual with a long supermaxilla.

Elemental compositions and stable isotope analysis of mature eggs revealed significant differences between individuals of the two ecotypes. The C:N ratios, *δ*^13^C and *δ*^15^N values of the anadromous individuals were 9.71 ± 0.22, −13.48 ± 1.36‰ and 11.58 ± 1.16‰, significantly higher than those of the freshwater-resident individuals (8.72 ± 0.34, −25.27 ± 0.98‰ and 8.16 ± 0.48‰, *U* = 0, *p* < 0.01, Mann–Whitney *U*-test). Scatter plot analysis indicated that the two ecotypes occupied distinct sections when considering both *δ*^13^C and *δ*^15^N values, with the freshwater-resident individuals in the lower left corner and the anadromous individuals in the upper right corner, showing clear differentiation with no overlap between individuals of the two ecotypes (e.g., *δ*^13^C values ranged from −15.30‰ to −10.58‰ in the anadromous individuals vs. −26.77‰ to −23.88‰ in the freshwater-resident individuals; *δ*^15^N values ranged from 10.16‰ to 13.11‰ in the anadromous individuals vs. 7.52‰ to 9.00‰ in the freshwater-resident individuals, Figure 3). Furthermore, the within-group CVs were 4.23% (freshwater-resident individuals) and 10.07% (anadromous individuals), and the between-group CV was 31.80% for *δ*^13^C; for *δ*^15^N, the within-group CVs were 5.90% (freshwater-resident individuals) and 9.99% (anadromous individuals), and the between-group CV was 19.82%. Meanwhile, based on the above stable isotope signatures, the cross-validation results of linear discriminant analysis (LDA) showed no misclassification between the two ecotypes, demonstrating strong discriminatory performance within the present dataset (Table 2).

## 4. Discussion

The Sr/Ca ratio of otoliths has been proven to be a crucial parameter for identifying the migration patterns and life-history characteristics of fish. Otolith microchemistry can also be used to reveal the migration characteristics of *C. nasus* and *C. mystus*, as well as the habitat history of the freshwater landlocked species *C. nasus taihuensis* [37]. Notably, the Sr/Ca (×1000) ratio in otoliths is commonly used as a discriminant indicator for different habitats (freshwater, brackish water, and marine water). For example, in *Anguilla* spp., the boundary values between these three habitat types are 2.5 and 6 [40], respectively; for *Chelon ramada*, these values are 2.89 and 8.63, respectively [41]. Notably, the previous study established identification criteria for freshwater, estuarine, and seawater habitats, corresponding to otolith Sr/Ca ratios of ≤3, 3–7, and ≥7, respectively, with color separation maps reflecting these regions (blue, yellow-green, and red); these characteristics were not affected by age or season [37]. Subsequent studies have confirmed this standard [2,25,36]. Meanwhile, it should be noted that although zones 3–7 are defined as estuarine/brackish water, they essentially encompass most of the coastal sea areas adjacent to the Yangtze Estuary. For instance, previous studies have found that for *C. nasus* individuals captured in sea areas approximately 200 km and 300 km from the Yangtze Estuary, the Sr/Ca ratios in their intermediate high-value zones were 4.44 ± 0.36 and 5.81 ± 0.66, respectively [42].

Our study revealed that the Sr/Ca ratios of adult *C. nasus* with short supermaxillae were stable and low (<3; Figure 2), typical of freshwater-resident individuals. In contrast, the Sr/Ca ratio of individuals with long supermaxillae exhibited three stages: a low-value zone in the core (<3), a high-value zone in the middle (3–7) with some individuals showing values exceeding 7 (e.g., CN01 and CN04; Figure 2), and a low-value zone at the edge (<3; Figure 2), which was characteristic of a migration pattern involving freshwater hatching, estuarine/brackish water, or seawater growth, and a final return to freshwater for reproduction. Therefore, for all 20 selected females, the morphological classification was consistent with the otolith microchemical classification. It indicated that individuals with short supermaxillae (CB) were considered freshwater-resident individuals, and those with long supermaxillae (CN) were considered typical anadromous individuals.

Subsequent stable isotope analysis of mature eggs demonstrated that both *δ*^13^C and *δ*^15^N values of the anadromous individuals were significantly higher than those of freshwater-resident individuals (Figure 3). Furthermore, based on the above stable isotope signatures, the cross-validation results of linear discriminant analysis (LDA) of mature eggs between the two ecotypes showed no misclassification, indicating strong discriminatory performance within the present dataset (Table 2). Similar results have been observed in *Salmo trutta* [38]. *C. nasus* do not feed during their migration until they reach their spawning grounds [28]. Therefore, the main substances for gonad development are directly linked to the feeding conditions in the estuary or sea. Rivers transport a large amount of terrigenous organic matter into the ocean, leading to significant differences in *δ*^13^C and *δ*^15^N values between marine and freshwater organisms [43]. These differences can be used to distinguish between freshwater and migratory populations [26,38]. In *S. trutta*, eggs from anadromous individuals had significantly higher *δ*^13^C (−19.9 ± 1.1‰) and *δ*^15^N (14.3 ± 1.5‰) values than those from freshwater-resident individuals (−25.7 ± 1.9‰ and 9.2 ± 1.8‰, respectively) [38]. The southern Yellow Sea is an important habitat of adult *C. nasus*. Notably, the main diet of *C. nasus* consisted of *Calanus sinicus* and *Leptochela gracilis* in the sea [44], and *Exopalaemon modestus* and *Macrobrachium nipponense* in the lakes [28], contributing to the *δ*^13^C and *δ*^15^N values observed in the present study. The *δ*^13^C and *δ*^15^N values of particulate organic matter in the Yellow Sea are −24.66 ± 0.69‰ and 6.42 ± 1.94‰, respectively [45], which are significantly higher than those in Poyang Lake (−27.2 ± 0.4‰ and 4.5 ± 0.4‰, respectively) [46]. Therefore, the higher *δ*^13^C and *δ*^15^N values observed in the mature eggs of the anadromous *C. nasus* in the present study are consistent with differences in feeding habitats (i.e., estuary, sea), even if these adults fed on swimming organisms (e.g., shrimp) to maintain sufficient energy before arriving at the spawning ground [28].

Given the difficulty of obtaining fully mature females with intact ovaries, a total of 102 individuals were collected but only 20 specimens were ultimately usable, resulting in a limited sample size. Nevertheless, the results showed that mature eggs from the two ecotypes differed markedly in *δ*^13^C and *δ*^15^N signatures, with no overlap between two ecotypes (e.g., normalized *δ*^13^C values ranged from −15.30‰ to −10.58‰ in the anadromous individuals vs. −26.77‰ to −23.88‰ in the freshwater-resident individuals; *δ*^15^N values ranged from 10.16‰ to 13.11‰ in the anadromous individuals vs. 7.52‰ to 9.00‰ in the freshwater-resident individuals; Figure 3). Moreover, within-group coefficients of variation (CVs) ranged from 5.90% to 9.99% for *δ*^15^N and from 4.23% to 10.07% for *δ*^13^C, which were much lower than the between-group CVs of 19.82% for *δ*^15^N and 31.80% for *δ*^13^C, indicating that these isotopic signatures can reliably discriminate mature eggs of the two ecotypes.

While the present study developed and validated the *δ*^13^C and *δ*^15^N discrimination technique specifically for mature eggs (collected from ovaries rather than from the field), the biochemical composition of fertilized eggs is largely inherited from the oocyte/egg. Therefore, our approach has considerable potential for distinguishing fertilized eggs of anadromous versus freshwater-resident *C. nasus*. It is important to note that all eggs were collected directly from ovaries and were unfertilized. After spawning and fertilization, eggs undergo water absorption and embryonic development, which may alter their isotopic composition through metabolic turnover, differential incorporation of maternal nutrients, or changes in tissue hydration. These processes could potentially shift *δ*^13^C and *δ*^15^N values and affect the discriminatory thresholds established here. Therefore, our results should be interpreted as evidence of maternal isotopic divergence and a proof-of-concept for egg-based discrimination, rather than as direct validation for field-collected fertilized eggs. Future studies should analyze naturally spawned fertilized eggs to determine whether the isotopic signatures remain stable and whether the classification criteria developed from ovarian eggs can be reliably applied in field settings. It should also be noted that the current baseline should be expanded with a larger sample of mature females from multiple spawning seasons and spawning grounds to capture the full range of natural variation before the technique is routinely applied to field-collected eggs.

Although lipid extraction was not performed, *δ*^13^C values were corrected for lipid content following the approach of Post et al. [39]. After lipid correction, *δ*^13^C values of mature eggs from the anadromous individuals remained significantly higher than those from the freshwater-resident individuals, and individuals of the two ecotypes did not overlap (e.g., corrected *δ*^13^C ranged from −15.30 to −10.58‰ in the anadromous individuals vs. −26.77 to −23.88‰ in the freshwater-resident individuals). This finding suggested that the observed differences in δ^13^C signatures were not driven solely by lipid content, and was consistent with distinct dietary carbon sources and migratory habitats. However, because a species-specific lipid correction equation has not yet been validated for *C. nasus* eggs, we cannot definitively attribute the remaining differences exclusively to dietary or habitat effects. In addition, *δ*^15^N values also differed significantly between the two ecotypes, further supporting the robustness of the discrimination. Given the extremely lipid-rich nature of mature eggs/embryos in *C. nasus* (total fatty acid content up to 418.95 mg/g [47]; total lipid up to 60.97% [48] of embryo dry weight), mathematical lipid correction was preferable to physical lipid extraction because it preserves sample quantity—an important advantage for future field applications, especially since *C. nasus* produces floating eggs that are difficult to collect. Finally, the marked differences between marine and freshwater habitats, as well as the contrasting migratory behaviors of the two ecotypes, likely contributed substantially to the observed isotopic divergence. Nevertheless, it is important to acknowledge that the equation of Post et al. [39] was developed from a dataset that included organisms with C:N ratios up to approximately 6.9, whereas the mean C:N ratios of our anadromous and freshwater-resident egg samples were 9.71 and 8.72, respectively—well above that calibration range. Consequently, the application of this model to our lipid-rich fish eggs was an extrapolation beyond its original scope. Therefore, the present lipid-corrected δ^13^C values should be treated as provisional approximations that reduce, but did not definitively eliminate, the confounding effect of lipids. Future studies should conduct physical lipid extraction and compare the results with mathematical corrections to develop a species-specific equation for *C. nasus* eggs, which would provide more reliable estimates. In addition, future studies should also compare specific biochemical fractions (e.g., proteins and fatty acids) of mature or fertilized eggs from both ecotypes using stable isotope analysis. Such comparisons would further refine our understanding of their isotopic divergence and nutritional characteristics.

## 5. Conclusions

Due to the unique feeding strategy and life history of the anadromous *C. nasus*, which only begins feeding upon reaching waters near their spawning grounds, *δ*^13^C and *δ*^15^N values of their mature eggs were significantly higher than those of the freshwater-resident individuals. The approach developed in this study, based on stable isotope analysis of mature eggs collected from ovaries, showed strong potential for distinguishing the two maternal life history types within the present dataset. However, because our analyses were performed exclusively on mature eggs and not on naturally spawned or fertilized eggs collected in the field, the technique has not yet been validated for identifying fertilized eggs or locating spawning grounds. Therefore, its application to spawning-ground localization should be considered preliminary and requires direct testing on field-collected fertilized eggs. Future studies should focus on collecting and analyzing naturally spawned eggs to evaluate this potential and to map the actual distribution of spawning grounds in Poyang Lake.

## Figures and Tables

**Table 1:** *Coilia nasus* sample details. CN: the individual with a long supermaxilla; CB: the individual with a short supermaxilla.

Sample Code	Body Length (mm)	Body Weight (g)	Supermaxilla Length/Head Length
CN01	309	105	1.13
CN02	328	114	1.16
CN03	264	62	1.15
CN04	337	110	1.24
CN05	263	61	1.25
CN06	293	89	1.15
CN07	314	102	1.12
CN08	317	102	1.12
CN09	328	113	1.18
CN10	343	127	1.21
CB01	302	103	0.99
CB02	285	93	0.94
CB03	297	103	0.97
CB04	327	124	0.96
CB05	278	78	0.99
CB06	326	128	0.98
CB07	269	65	0.97
CB08	319	108	0.97
CB09	306	109	0.97
CB10	249	67	0.98

**Table 2:** Classification of stable isotope of *Coilia nasus*, performed using linear discriminant analysis. CB: the individual with a short supermaxilla; CN: the individual with a long supermaxilla.

Ecological Type	Predicted Group (Original/Cross-Validated)		Correctly Classified % (Original/Cross-Validated)
CB (*n* = 10)	10/10	0/0	100/100
CN (*n* = 10)	0/0	10/10	100/100

## Data Availability

Data will be made available on request.

## Abbreviations

The following abbreviations are used in this manuscript:

air N2	Atmospheric nitrogen
CB	The individual with a short supermaxilla
CN	The individual with a long supermaxilla
VPDB	Vienna PeeDee Belemnite
LDA	linear discriminant analysis
CV	Coefficient of variation
